# Unsupervised feature extraction using deep learning empowers discovery of genetic determinants of the electrocardiogram

**DOI:** 10.1186/s13073-025-01510-z

**Published:** 2025-10-09

**Authors:** Ewa Sieliwonczyk, Arunashis Sau, Konstantinos Patlatzoglou, Kathryn A. McGurk, Libor Pastika, Prisca K. Thami, Massimo Mangino, Sean L. Zheng, George Powell, Lara Curran, Rachel J. Buchan, Pantazis Theotokis, Nicholas S. Peters, Bart Loeys, Daniel B. Kramer, Jonathan W. Waks, Fu Siong Ng, James S. Ware

**Affiliations:** 1 National Heart and Lung Institute, Imperial College London, London, SW3 6LY UK; 2 MRC Laboratory of Medical Sciences, Imperial College London, Du Cane Road, London, W12 0HS UK; 3 University of Antwerp and Antwerp University Hospital, Antwerp, Belgium; 4 Department of Cardiology, Imperial College Healthcare NHS Trust, London, UK; 5 Department of Twin Research and Genetic Epidemiology, King’s College London, London, UK; 6 Royal Brompton and Harefield Hospitals, Guy’s and St. Thomas’ NHS Foundation Trust, London, UK; 7 Harvard-Thorndike Electrophysiology Institute, Beth Israel Deaconess Medical Center, Harvard Medical School, Boston, MA USA; 8 Chelsea and Westminster Hospital NHS Foundation Trust, London, UK

**Keywords:** GWAS, ECG, Deep learning, PheWAS, Burden testing, Variational autoencoder

## Abstract

**Background:**

Electrocardiograms (ECGs) are widely used to assess cardiac health, but traditional clinical interpretation relies on a limited set of human-defined parameters. While advanced data-driven methods can outperform analyses of conventional ECG features for some tasks, they often lack interpretability. Variational autoencoders (VAEs), a form of unsupervised machine learning, can address this limitation by extracting ECG features that are both comprehensive and interpretable, known as latent factors. These latent factors provide a low-dimensional representation optimised to capture the full informational content of the ECG. The aim of this study was to develop a deep learning model to learn these latent ECG features, and to use this optimised feature set in genetic analyses to identify fundamental determinants of cardiac electrical function. This approach has the potential to expand our understanding of cardiac electrophysiology by uncovering novel phenotypic and genetic relationships.

**Methods:**

Our novel VAE model was trained on a dataset comprising over one million secondary care median beat ECGs, with external validation in the UK Biobank (UKB). We performed common and rare variant association studies for VAE latent factors and conventional ECG traits on quality-controlled UKB data. Associated genetic variants were compared to loci for conventional ECG parameters available in the UKB and literature. Loci were considered novel if they were not previously associated with ECG traits in the GWAS Catalog and showed no known associations in nearby genes based on literature review. Novel GWAS associations were validated in a withheld subset of the UKB cohort. Additionally, we compared the associations of the VAE latent factors and conventional ECG traits with phenotypic traits, disease codes, and echocardiographic traits.

**Results:**

The VAE identified 20 independent latent factors that captured ECG morphology with high accuracy (mean Pearson correlation: 0.95). GWAS of latent factors identified 65 unique loci, including 27 novel regions not associated with conventional ECG parameters in the same dataset. Six novel loci were not associated with the ECG in previous larger GWAS studies, including genes implicated in cardiac function and remodelling. Rare variant analysis identified seven additional genes with links to cardiac electrophysiology and remodelling. Phenotypic analyses revealed stronger and more comprehensive associations for latent factors compared to conventional traits, particularly for echocardiographic measures and cardiac phenotypes. Visualisations of latent factor alterations highlighted the interpretability of this approach.

**Conclusions:**

Our study shows that the VAE provides a valuable tool for advancing our understanding of cardiac function and its genetic underpinnings, outperforming traditional approaches in genetic and phenotypic discovery.

**Supplementary Information:**

The online version contains supplementary material available at 10.1186/s13073-025-01510-z.

## Background

The application of machine learning techniques to understand cardiac electrophysiology has sharply risen in recent years, particularly in their application to the electrocardiogram (ECG) [1, 2]. Many of these applications rely on supervised models, which are designed to identify specific pre-defined diagnoses or predict outcomes based on the raw ECG signal as input. Apart from features directly related to cardiac function, machine learning-based models have been remarkably successful at deducing more general characteristics from the ECG, such as age and BMI [3–5].

Despite their impressive performance, the inner workings of these models are obscure, contributing to their reputation as a “black box”. This lack of transparency and interpretability limits the potential to generate novel biological insights. More interpretable unsupervised models, such as the variational autoencoder (VAE), have been applied to the ECG [6] as an alternative approach.

The VAE combines principles derived from machine learning and Bayesian inference to deconstruct a signal into a limited number of highly informative features, also called latent factors (LF). These features are optimised to capture the extent of inter-sample variability and can be used for signal generation [7], denoising [8] or predictive and diagnostic models [6]. The features extracted by the VAE are interpretable and lend themselves easily to visualisation, enhancing the model’s transparency and facilitating a deeper exploration of the underlying data dynamics.

Most conventional human-defined ECG features have emerged as patterns that are easy for human observers to recognise and label, and then correlate with clinical significance. By contrast, VAE latent factors are optimised to maximise the capture of data content in the ECG, without being constrained by human optical recognition or by prior knowledge and biases. We hypothesised that analysis of the genetic and phenotypic associations of this optimised feature set would empower discovery when compared with analysis of conventional ECG features, and therefore yield novel insights into cardiac electrophysiology and related diseases.

## Methods

### Datasets

This study used two primary cohorts for model training, genetic association, and replication analyses: the Beth Israel Deaconess Medical Center (BIDMC) cohort and the UK Biobank (UKB) [9].

The BIDMC cohort includes routinely collected ECG data from patients aged > 16 years (median age 65 years ± 16 SD at the time of examination) at BIDMC (Boston, USA), a tertiary medical centre. Subjects with a valid digital 10-s ECG recorded between 2014 and 2023 were included. Disease phenotypes were derived from ICD codes in the electronic health record; the dataset was supplemented by tabular echocardiographic data derived from the clinician’s report. This cohort was used primarily for model training (1,048,778 ECGs), internal validation (58,265 ECGs) and out-of-sample performance testing (58,268 ECGs). A subset of the dataset was used for phenotypical association studies (176,536 ECGs for training and 9282 in the test set) (Additional File 1: Fig. S1).

The UKB is a large prospective population-based study comprising over 500,000 individuals enrolled between 2006 and 2010 across the UK, aged 40–69 at baseline. Participants provided health and lifestyle information, underwent physical examinations, and contributed biological samples. A subset underwent follow-up visits with enhanced phenotyping, including digital ECG acquisition. For this study, we included 31,118 individuals with high-quality digital ECGs recorded during the second (“instance 2”) imaging visit for latent factor GWAS. An additional 18,987 participants from a later imaging visit were used for replication analyses. Additionally, a training subset of 32,832 ECGs and a testing subset of 8208 ECGs was used for phenotypic association studies (Additional File 1: Fig. S1). Data were accessed with approvals in place (Application numbers 47602 and 48,666) [10].

Genotyping was performed by the UKB central team with the Applied Biosystems (Affymetrix) UK BiLEVE Axiom Array or the UKB AxiomTM Array [10]. Imputation was done with the Haplotype Reference Consortium and the merged UK10K and 1000 Genomes phase 3 (1000G) reference panels [10].

### Median ECG derivation

All median beat ECGs were obtained from resting 10-s ECG signals with BRAVEHEART ECG analysis software (https://github.com/BIVectors/BRAVEHEART) [11]. The median beat was further adjusted by cropping or zero-padding to a length of 0.8 s (sampled at 500 Hz) and aligning the signals by cross-correlation. In order to remove signals with large pacing spikes or excessive noise, signals with extreme voltages (< − 8 mV or > 8 mV) were excluded from the analysis. After median ECG derivation and filtering, the remaining 1,165,311 median ECGs were randomly split into a training set (1,048,778 ECGs from 180,679 individuals), a validation set (58,265 ECGs from 4233 individuals) and a test set (58,268 ECGs from 4500 individuals), in a 90/5/5% split.

### VAE architecture

In order to extract ECG features, we employed a convolutional architecture due to its ability to efficiently capture localised temporal patterns in high-resolution ECG signals. The VAE framework, in contrast to a standard autoencoder, ensures that the learned latent space follows a continuous, structured distribution, which was essential for downstream analyses such as GWAS.

The VAE consists of three components: the encoder, the decoder and the latent space. The encoder and the decoder are made up of one-dimensional convolutional layers with increasing filters and decreasing kernel sizes closer to the latent space. The latent space was restricted to 30 features at a maximum, although typically only a subset of these features was used by the model for the reconstruction. The model was trained to minimise both the median ECG reconstruction loss, defined by a symmetric mean absolute percentage error function, and the Kullback–Leibler divergence (KL loss). This second term is specifically added to the VAE model to ensure that the features generated by the model are generative and disentangled. An additional β-parameter was included as a weight on the KL-term to optimise the balance between the reconstruction loss and the latent factor interpretability. We tested beta values of 0.1, 0.25, 0.5, 1, 3, 5, and 10 and defined the best model at a β-parameter of 0.25 based on the Pearson correlation between the median and its reconstruction in the validation dataset, as well as a visual inspection of the latent vector traversals.

### GWAS and common variant gene analysis

The UKB participants included in the genetic analyses were selected for European ancestry, missingness rate of SNPs < 10%, no sex discrepancies, and removing outliers of heterozygosity or relatedness. After selection, ECGs were available for 31,118 individuals. Quality control was performed to exclude SNPs with a minor allele frequency < 0.1%, genotyping rate < 95%, deviation of heterozygosity with Hardy–Weinberg equilibrium *p* < 1.0 × 10^−8^ or < 0.4 INFO imputation score.

The GWAS was carried out with the FastGWA MLM implemented by the Genome-wide Complex Trait Analysis (GCTA) software using a genetic relationship matrix (GRM) to adjust for population structure [12]. The latent factor distributions were normalised by rank-based inverse normal transform prior to the analysis. Age, sex, height, BMI, the UKB assessment centre, and the first 10 genetic principal components were included as covariates. We report both the SNPs which were identified by the conventional genome-wide significance threshold, with Bonferroni correction for testing of multiple features (*p*-value < 5 × 10^−8^/20 for the LF and < 5 × 10^−8^/11 for the traditional ECG parameters). A less stringent threshold at < 1% false discovery rate (FDR) was applied to the LF GWAS to select loci which were not previously associated with the ECG in the GWAS catalog.

The genetic variance explained by genome-wide SNPs (SNP-based heritability) was calculated with the genomic-relatedness-based restricted maximum likelihood (GREML) analysis using the GCTA software [13]. Genetic correlation was calculated with the bivariate GREML analysis method [14].

The common variant gene analysis was performed with MAGMA software on the summary statistics obtained from the GWAS [15]. We used 18,383 genes with a genome-wide significance threshold defined as *P* = 2.72 × 10^−6^.

### Locus identification and finemapping

Conditionally independent genetic variants were identified using a chromosome-wide stepwise conditional-joint analysis implemented in the GCTA software [16]. Variants within 500 kb of each other were aggregated and an additional 500 kb were added to flank the variants on each side of the locus. A locus was considered novel if it was not previously associated with electrocardiographic traits according to the GWAS catalogue. Potential novel loci were further validated by a literature search of nearby genes.

Functionally informed fine mapping with PolyFun [17] and SuSiE [18] was performed to identify the most likely causal variants. Precomputed prior causal probabilities from the PolyFun UKB analysis were used to compute the per-SNP heritability. Linkage disequilibrium was calculated for each locus and used for fine-mapping. Using SuSiE, we calculated the per-SNP posterior inclusion probability (PIP) and identified 95%-credible sets of likely causal variants, under the assumption of at most 5 causal variants for each locus. These variants were used for annotation with the nearest protein-coding gene and gene prioritisation. As several regions did not contain 95%-credible sets of likely causal variants for the regions identified by the 5% FDR threshold, SNPs with the lowest *p*-values were used instead.

### Gene prioritisation

Candidate genes were selected using three approaches: nearest gene annotation, the polygenic priority score (PoPS) [19] and variant-to-gene (V2G) [20]. PoPS uses gene expression, biological pathways and protein-protein interactions to assign priority to genes, based on their similarity to other potentially causal genes identified based on a prior functional annotation and gene-based analysis of the GWAS summary statistics by the MAGMA software [15]. Genes outside the GWAS loci which were significantly associated with the phenotype according to the MAGMA analysis were reported separately. The V2G method is based on the integration of epigenomic data (eQTL, pQTL, sQTS, chromatin interactions), functional predictions and genomic distance to assign a variant level score, with higher scores representing a higher likelihood of a functional effect on a target gene. The candidate list was generated by selecting the top three highest scoring genes for both the PoPS and V2G methods, as well as the MAGMA significant genes and the nearest protein coding gene for each region (Additional File 2 - GWAS regions).

To prioritise the most likely causal gene for each locus, we first selected all established Mendelian cardiac arrhythmia [21] and cardiomyopathy [22] genes out of the candidate list. Secondly, we selected the genes which were selected by > 1 method (PoPS, V2G and MAGMA). If several genes were selected by an equal number of methods at one locus, a literature search was conducted to identify the gene with the strongest relationship to cardiac function. If multiple genes were plausible, we selected all candidates for the region. If there was no evidence for any of the genes in literature, we selected the nearest gene.

### Pathway analysis

The GWAS prioritised genes were assessed for tissue specificity in the GTEx v7 (30 general tissue types) and gene set enrichment for molecular function Gene Ontology terms, with all protein coding genes as background, with Functional Mapping and Annotation of Genome-Wide Association Studies (FUMA) software [23].

### Validation of novel GWAS hits

We performed GWAS validation in a separate cohort of UKB digital ECGs collected more recently, which became available at the time of writing this manuscript. This dataset consists of ECGs from 24,355 participants, 18,987 of which were included in the GWAS validation cohort after quality control. As the lower sample size resulted in a lack of model convergence for some LF with the fastGWA-REML (GCTA) analysis, the validation cohort GWAS was done with REGENIE software [24]. Variants from the LF GWAS significant at the FDR < 1% threshold, which were not previously associated with ECG traits, were selected for validation. Variants with a p-value of less than 0.05 in the validation cohort were reported as novel findings.

### Rare-variant gene-based association testing

We performed rare-variant gene-based association testing for each normalised latent factor with the UKB 500 k whole-exome sequencing (WES) data using the REGENIE software [24]. As a first step, a regression model was fitted to correct for polygenicity, ancestry, and relatedness, with the SNPs which were previously selected for the GWAS analysis. In the second step, we performed exome-wide gene-based burden testing, with a significance threshold defined at false discovery rate (FDR) < 5%. Covariate adjustment for sex, age, age^2^, height, BMI, and the 10 first genetic principal components was included. Masks were constructed based on variant frequency (singleton, < 0.001 and < 0.01) and likelihood of a pathogenic effect. For missense variants, this was determined by a pathogenicity score based on a combination of five prediction tools (SIFT, PolyPhen2 HDIV, PolyPhen2 HVAR, LRT and MutationTaster) [25]. As previously published, variants which were predicted deleterious by all five algorithms were considered as “likely deleterious” [25]. Only loss-of-function and likely deleterious missense variants were included in the analysis. We initially considered 18,985 protein coding genes with subsequent subset analysis focusing on the prioritised genes from the GWAS analysis, with the FDR-corrected *p*-values adjusted to the number of genes for each analysis.

### Phenotypic associations

Phenotypic association studies were performed in the BIDMC for binary disease phecodes (*n* = 2074) and echocardiographic traits (*n* = 39), and in the UKB for continuous phenotype traits (*n* = 2093). We developed two separate regression models, one with all the LFs as predictors and one with the available ECG parameters as predictors and age, age^2^, and sex as covariates. The BIDMC ECG parameters were heart rate, QRS duration, QT/QTc interval, and PR interval; for the UKB, P axis, PQ interval, QTc interval, QT interval, QRS duration, Ventricular Rate, PP interval, QRS number, P wave duration, R axis, and T axis were used.

The models were used to obtain phenotype predictions, which were correlated with the actual phenotype values (biserial correlation coefficient for binary traits and Pearson’s correlation coefficient for continuous traits). Correlations with a Bonferroni corrected *p*-value below the 0.05 threshold were excluded from the analysis.

For the BIDMC dataset, the models were developed with ECGs from the VAE training dataset; predictions were derived from ECGs in the test set. We excluded phenotypes with > 90% missing values and binary phenotypes with counts < 100 in the training set. For the disease phecodes, we used a single random ECG per subject (*n* = 176,536 for training, 9282 for predictions). For the echocardiographic traits, we used a single ECG per subject, taken < 30 days before or after the echocardiography (*n* = 58,063 for training, 3006 for predictions). The use of a single ECG per subject was necessary because the regression models employed in these analyses are not designed for datasets containing repeated measurements from the same individual. For the UKB dataset, we randomly split the dataset into a training (*n* = 32,832) and a test set (*n* = 8208).

## Results

### Accurate median ECG reconstructions with 20 novel independent ECG features

The VAE model architecture is displayed graphically in Additional File 1: Fig. S1. The model was trained on 1,048,778 median ECG beats derived from resting 12-lead ECGs of patients examined at the Beth Israel Deaconess Medical Center (BIDMC), a secondary care centre in Boston, USA. Eight leads were analysed—the first two limb leads and six chest leads. Lead III and the augmented limb leads were not used as they are linear combinations of leads I and II and therefore provide no additional information. Hyperparameter tuning was performed using the validation set (*n* = 58,265 ECGs) and performance was evaluated in the test set (*n* = 58,268 ECGs). External validation was performed on digital ECG recordings from the UK Biobank (UKB) (*n* = 42,248). Median beats were extracted using the Beth Israel Analysis of Vectors of the Heart (BRAVEHEART) software as previously described [11]. Further details on these datasets are available in the Supplementary Materials (Additional File 1: ECG datasets section).

**Fig. 1:**
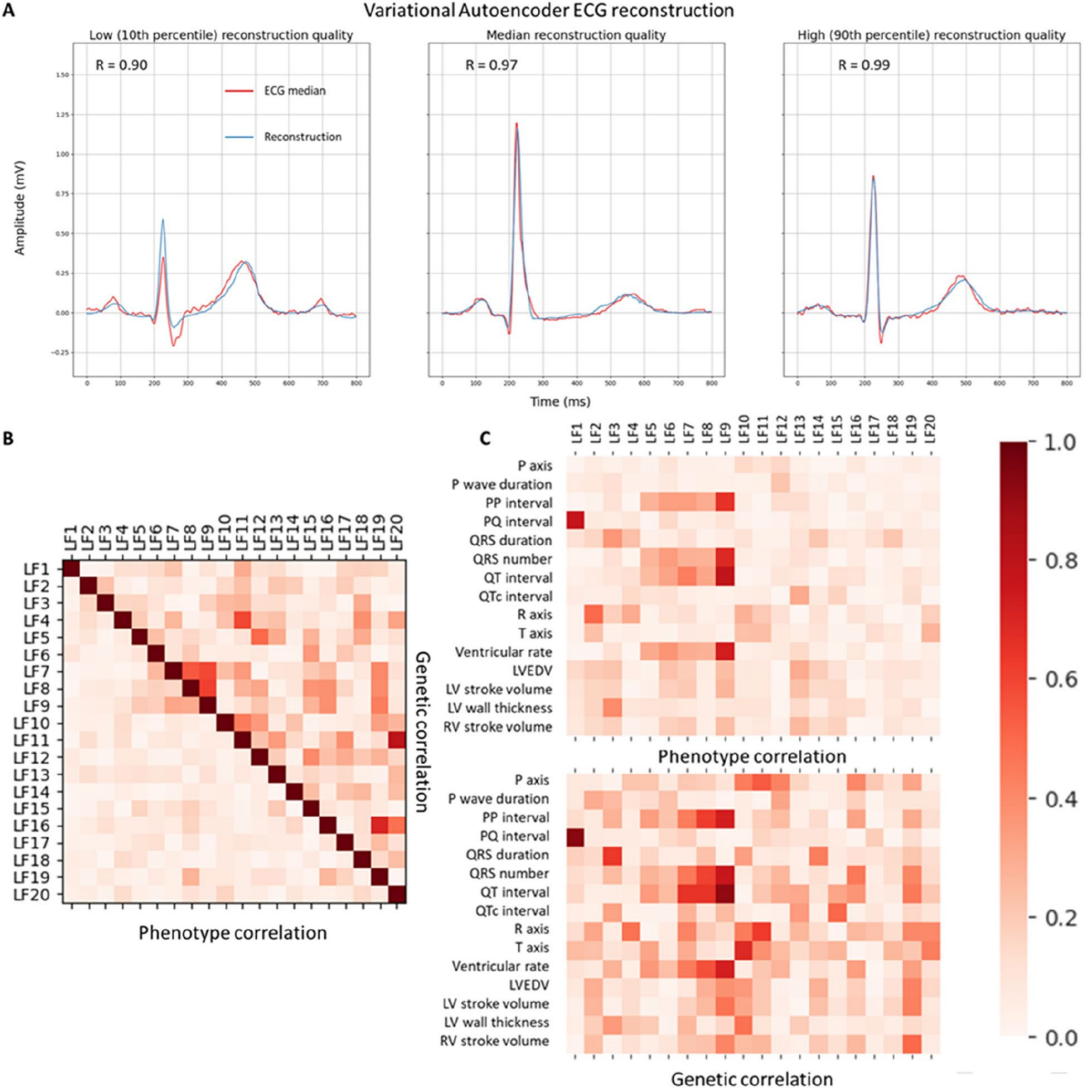
Variational autoencoder accuracy and latent factor correlations. **A** The Variational autoencoder produces precise median beat reconstructions (blue) that closely match the original ECG medians (red). Example reconstructions spanning a range of accuracies are shown, by sampling reconstructions at the 10th percentile, median, and 90th percentile of the correlation distribution. **B** A heatmap illustrates the absolute phenotypical correlations (Pearson’s *R*, bottom left) and absolute pairwise genetic correlations (rg, SNP-based heritability, top right) between individual latent factors in the UKB, indicating phenotypically uncorrelated novel ECG features with a shared genetic architecture. **C** The heatmaps depict the absolute phenotypical correlations (Pearson’s *R*, top) and genetic correlations (rg, bottom) between latent factors, traditional ECG features and structural cardiac features (based on cardiac MRI analysis) in the UKB. LV left ventricle, RV right ventricle, EDV end diastolic volume)

When comparing the reconstructions to the original medians, we observed an excellent mean Pearson correlation coefficient of 0.94 (± 0.11 SD) in the (BIDMC) test set and 0.95 (± 0.12 SD) in the UKB. As demonstrated in Fig. 1A, the reconstructions accurately captured the typical ECG morphology.

**Fig. 2:**
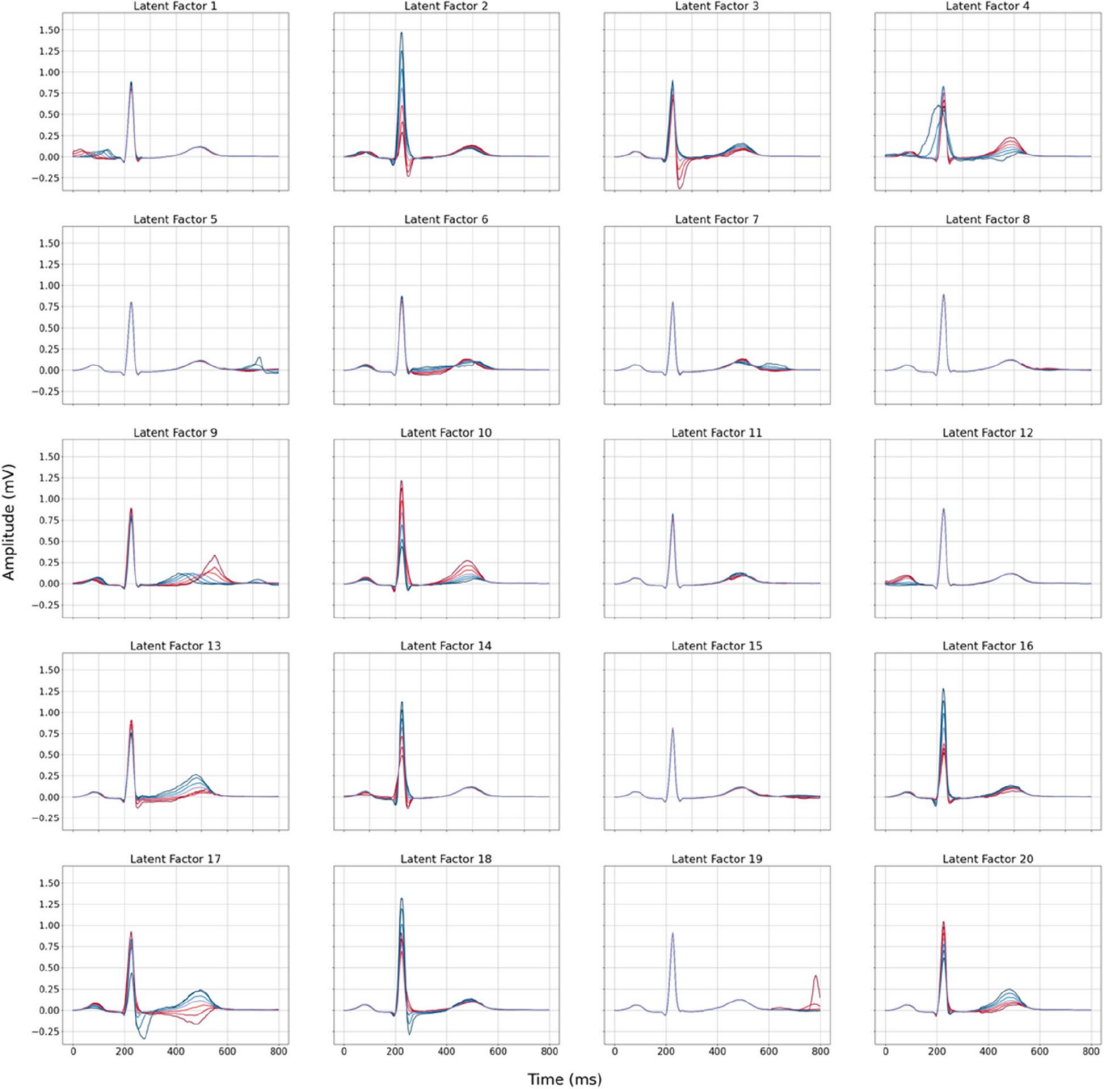
Lead I latent traversal plots of the 20 latent factors identified by the Variational Autoencoder demonstrate independent encoding of ECG components. Each plot corresponds to the reconstruction of the median beat in Lead I, exploring the influence of a single latent factor (range: −3 to +3 standard deviations from the mean), while maintaining others at the mean value. Red lines indicate negative deviations, while blue lines indicate positive deviations

Each LF was visualised by modifying that specific LF (at a range of ± 1–3 SD of the LF mean as calculated in the BIDMC test set) while holding the others at their mean value (latent traversal, Fig. 2). The latent traversal indicated that different LFs were representative of independent ECG traits. This was further confirmed by the lack of phenotypic correlation between the individual LFs (Fig. 1B), with only two LFs showing an absolute Pearson correlation coefficient > 0.3 (LF 7 and 9, *R* = 0.31). Full 8-lead latent traversals, as well as an interactive plot of the LFs, are available in the Supplementary Materials (Digital ECG Explorer App, ECG Latent Factor Explorer).

To explore the relationship between the novel LFs, traditional ECG parameters and several disease-relevant structural cardiac traits, we calculated the Pearson correlation coefficient between the LFs and the available ECG metrics in the UKB (P axis, PQ interval, QT interval, QTc interval, QRS duration, ventricular rate, PP interval, number of QRS complexes during the 10-s ECG (QRS number), P wave duration, R axis and T axis), as well as selected cardiac MRI traits from the UKB (left and right ventricle stroke volume, left ventricle wall thickness and left ventricle end diastolic volume).

Most of the LFs capture information not represented in the traditional ECG features, as evidenced by their weak correlation with traditional ECG parameters, with only seven LFs displaying an absolute Pearson correlation coefficient above 0.3 with any ECG parameter (LFs 1–3 and 6–9, Fig. 1C). These seven LFs accounted for most of the associations with the traditional ECG parameters, though three of these ECG features (P-axis, P wave duration, T-axis) displayed a low (Pearson *R* < 0.3) correlation with the LFs. The highest correlation was observed for the QT interval with LF9 and the PQ interval with LF1 (Pearson *R* 0.78 and 0.77, respectively).

### The genetic architecture of the VAE latent factors

Overall, the LFs showed a variable degree of heritability, ranging from 3 to 20% (Supplementary Materials – Digital ECG Explorer App, ECG to Gene). Despite the lack of phenotypical correlation, several LFs proved to be genetically correlated with each other (absolute rg between 0.0003 and 0.79; Fig. 1B). Similarly, we observed higher values for genetic correlations with the traditional ECG features and structural cardiac traits than the phenotypical correlations. Only three LFs showed a pairwise genetic correlation coefficient of less than 0.3. All genetic correlations between ECG parameters and LFs were above 0.3 (Fig. 1C).

To assess the specificity of these correlations and provide a negative control, we also evaluated genetic correlations between the 20 LFs and age—a quantitative trait that is not genetically determined. Although some potential confounding due to survivorship bias cannot be excluded, age was selected as it is unlikely to share substantial heritable components with ECG morphology. In contrast to the ECG traits, the correlations with age were markedly lower: only two LFs (LF10 and LF20) showed an absolute genetic correlation > 0.3, compared to 220 out of 220 LF–ECG trait correlations exceeding this threshold (Additional File 1: Table S1).

These findings are indicative of the presence of pleiotropic genetic factors which act on multiple phenotypically uncorrelated ECG features. The associations of specific loci with ECG morphology are shown through locus-specific LF traversals in the Supplementary Materials (Digital ECG Explorer App, Gene to ECG).

**Fig. 3:**
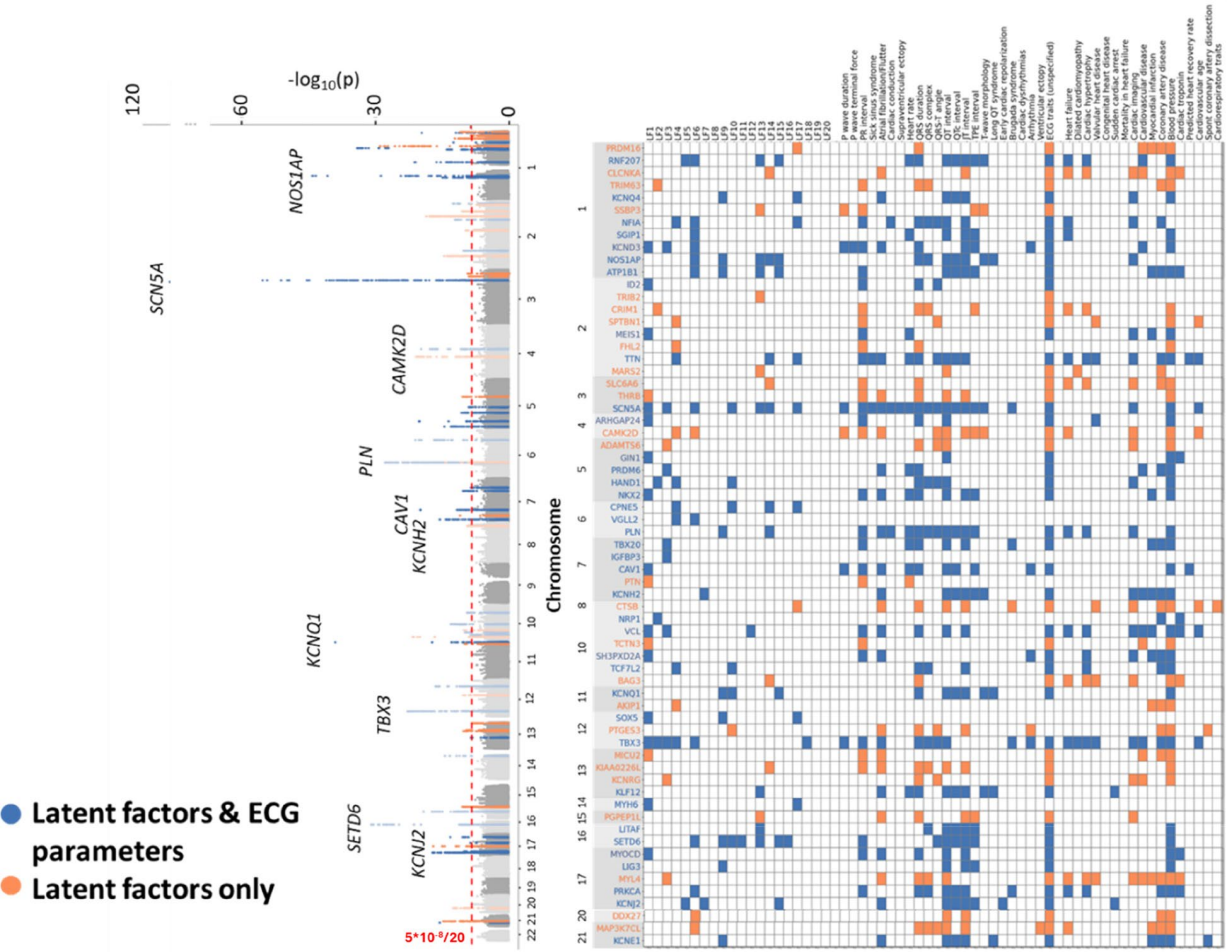
Latent factor GWAS joint Manhattan plot, with prioritised genes and their relationship to previous GWAS hits for ECG parameters and cardiac function-related traits. Left side: the Manhattan plot collapses the latent factor (LF) traits into one plot by displaying the lowest *p*-value among the 20 LF GWAS for each of the included SNPs, calculated in the UK biobank. Right side: First listed prioritised gene (full gene list is available in Additional File 2 - GWAS regions) with their associated LF, previously reported ECG features, and cardiac function-related traits. The colours represent the comparison between the LF GWAS and traditional ECG parameters GWAS (including P axis, PQ interval, QTc interval, QT interval, QRS duration, Ventricular Rate, PP interval, QRS number, P wave duration, R axis and T axis). Blue: regions which are identified by the LF GWAS and the ECG parameter GWAS in our study and have been previously validated in other GWAS analyses of ECG traits. Orange: regions which are identified by LF GWAS only in our study (not conventional ECG traits), but have been previously validated in other GWAS analyses of ECG traits

GWAS analysis of the 20 LFs identified 120 conditionally independent SNPs, corresponding to 118 genomic regions (*P*_adj_ < 2.5 × 10^−9^) (Additional File 3 - conditional SNPs). After combining regions with overlapping borders, we identified 65 unique loci that were associated with one or more LFs. Prioritised genes were mapped to the GWAS regions by a combination of similarity-based (polygenic priority score or PoPS) [19] and locus-based (variant-to-gene or V2G) [20] approaches, yielding 85 genes (Fig. 3) [15]. The LFs were able to capture more significantly associated genes when compared to the analysis of traditional ECG parameters on the same dataset. Traditional parameters were associated with 93 conditionally independent SNPs, corresponding to 51 unique regions that were associated (*P*_adj_ < 4.5 × 10^−9^) with one or more traditional ECG parameters. Twenty-seven (42%) of the LF loci were not detected by GWAS of ECG parameters, while 13 (25%) of the regions identified by the analysis of ECG parameters were not identified by the LFs. These regions were associated with 9 unique genes, all of which had been previously reported for these traits in the GWAS catalog (see Additional File 4 - GWAS regions where traditional ECG parameters identified loci missed by the latent factors).

### Identification and validation of novel genomic regions associated with the ECG

In order to identify novel common variant associations with the ECG, we identified LF GWAS loci not linked to any ECG traits in the GWAS catalog. One locus (mapped to *NRP1*) qualified. For discovery purposes, we additionally evaluated a broader set of genes at a more relaxed threshold of < 1% FDR. We identified 46 loci which associated with any of the LF and were not previously linked to the ECG. We compensated for the increased risk of false positive associations due to the relaxed significance threshold by reanalysing these candidate SNPs in a separate tranche of data from 18,987 UKB participants, which was released after the main analysis. We identified six SNPs which were directionally concordant and significant (p-value < 0.05) in the validation tranche. These loci were mapped to the genes *NRP1, TRIOBP, EFEMP1, NEDD9, GPC6,* and *SEC14L4.* Two of these six loci, mapped to *NRP1* and *NEDD9*, were also associated with traditional ECG parameters at the same significance threshold.

### Pathway analysis

**Fig. 4:**
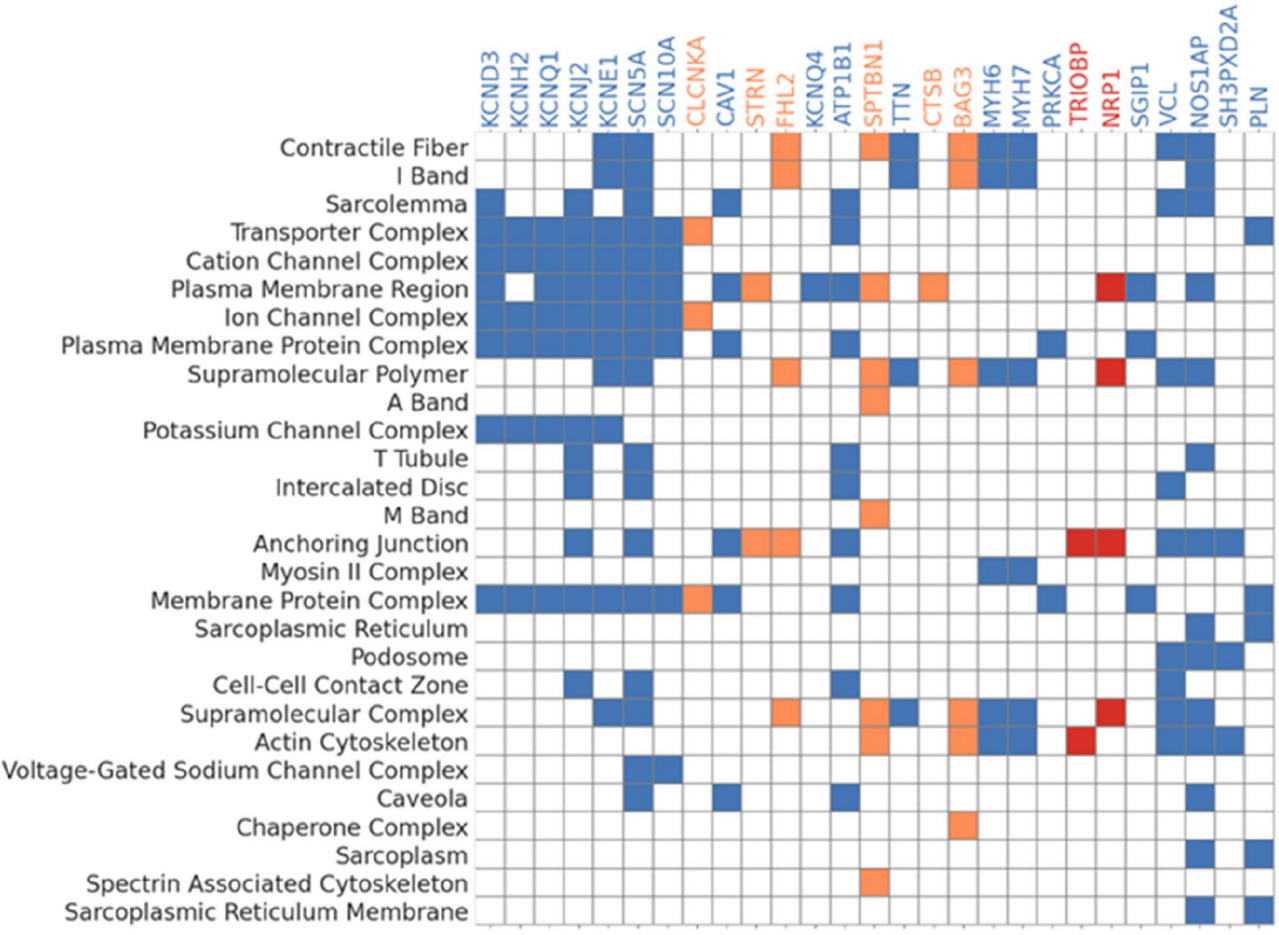
Significantly enriched Gene Ontology Cellular Components terms and the associated prioritised genes in the latent factor (LF) GWAS. Analysis of enriched pathways demonstrates shared molecular functions for genes identified by analysis of both traditional ECG parameters and LF, as well as the LF only and our novel findings. For visualisation purposes, individual genes are shown in relation to the enriched GO terms they are annotated to, but the enrichment statistics are computed at the gene set level. Blue: regions which are identified by the LF GWAS and the ECG parameter GWAS in our study and have been previously validated in other GWAS analyses of ECG traits. Orange: regions which are identified by LF GWAS only in our study and have been previously validated in other GWAS analyses of ECG traits. Red: novel regions, which have not been linked to the ECG in previous GWAS but have a p-value <1% in the LF GWAS and replicate in a hold-out validation tranche

We performed gene function analysis for all the prioritised GWAS genes (*n* = 85) with Functional Mapping and Annotation of Genome-Wide Association Studies (FUMA) software [23]. All protein-coding genes were considered as the background genes. The prioritised genes were more highly expressed in the heart (*P*_adj_ 2.16 × 10^−8^) and blood vessels (*P*_adj_ 0.001). The analysis of gene-set enrichment, focusing on cellular components Gene Ontology terms, revealed 28 terms that exhibited significant enrichment within our gene list (Fig. 4). Most of these terms were related to cardiac ion channels, the sarcomere, and the cytoskeleton.

### Gene-wise rare variant association study reveals additional genes associated with latent factors, and provides support for LF GWAS loci

**Fig. 5:**
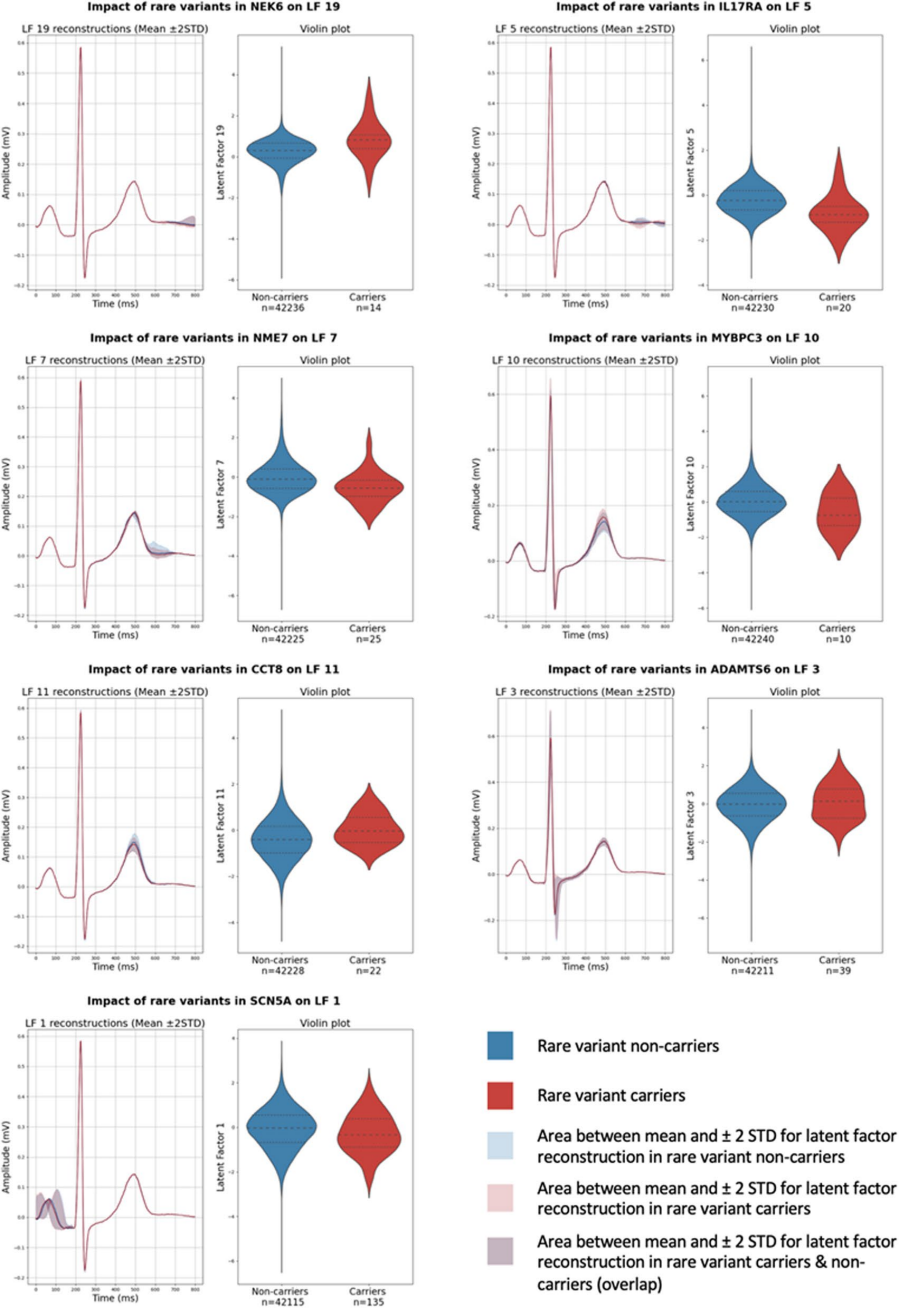
ECG median reconstructions and violin plots for carriers and non-carriers of rare variants associated with the latent factors. The median reconstructions were generated by holding all latent factors as a set value and altering the significantly associated latent factor. Dashed lines: medians, dotted lines: quartiles. The lines represent the reconstructions at the population mean and the shaded area indicating ± 2 standard deviations

The rare variant analysis was performed by gene burden testing on the UKB cohort (*n* = 32 670 ECGs), initially through an exome-wide approach, with subsequent subset analysis focusing on the prioritised genes from the GWAS (*n* = 85). With the whole-exome approach, we identified two genes, which demonstrated significant rare-variant associations with the LFs: *NEK6* with LF19 and *IL17RA* with LF5 (both for singleton protein-altering variants, Fig. 5). Subsequent subset analysis focusing on the prioritised genes identified five additional associations (Fig. 5). Singleton variants in the *NME7* gene were associated with LF7. LF10 was associated with variants in *MYBPC3* with an allele frequency < 0.001. Rare (allele frequency < 0.001) variants in *CCT8* were associated with changes in LF11. Surprisingly, in the GWAS analysis, this locus was associated with LF7, rather than LF11. Low frequency (< 0.01) variants in the *ADAMTS6* gene were associated with LF3. Finally, variants in *SCN5A* (< 0.01 allele frequency) were associated with LF1 (Fig. 5), which is closely correlated to the PQ interval.

### Phenotypic associations with the latent factors

To identify phenotypic associations, we developed separate regression models with the 20 LFs or the available ECG parameters as predictors and age, age^2^, and sex as covariates. The models were used to obtain phenotype predictions in the test set, which was entirely separate from the training dataset, which was withheld from the dataset used to train the regression models. These predictions were correlated with the actual phenotype values (biserial correlation coefficient for binary traits and Pearson’s correlation coefficient for the continuous traits). We tested the correlation for 2074 binary disease phecodes in the BIDMC, 2093 continuous phenotype measures in the UKB, and 39 echocardiographic traits in the BIDMC. Bonferroni correction was applied to account for multiple testing.

**Fig. 6:**
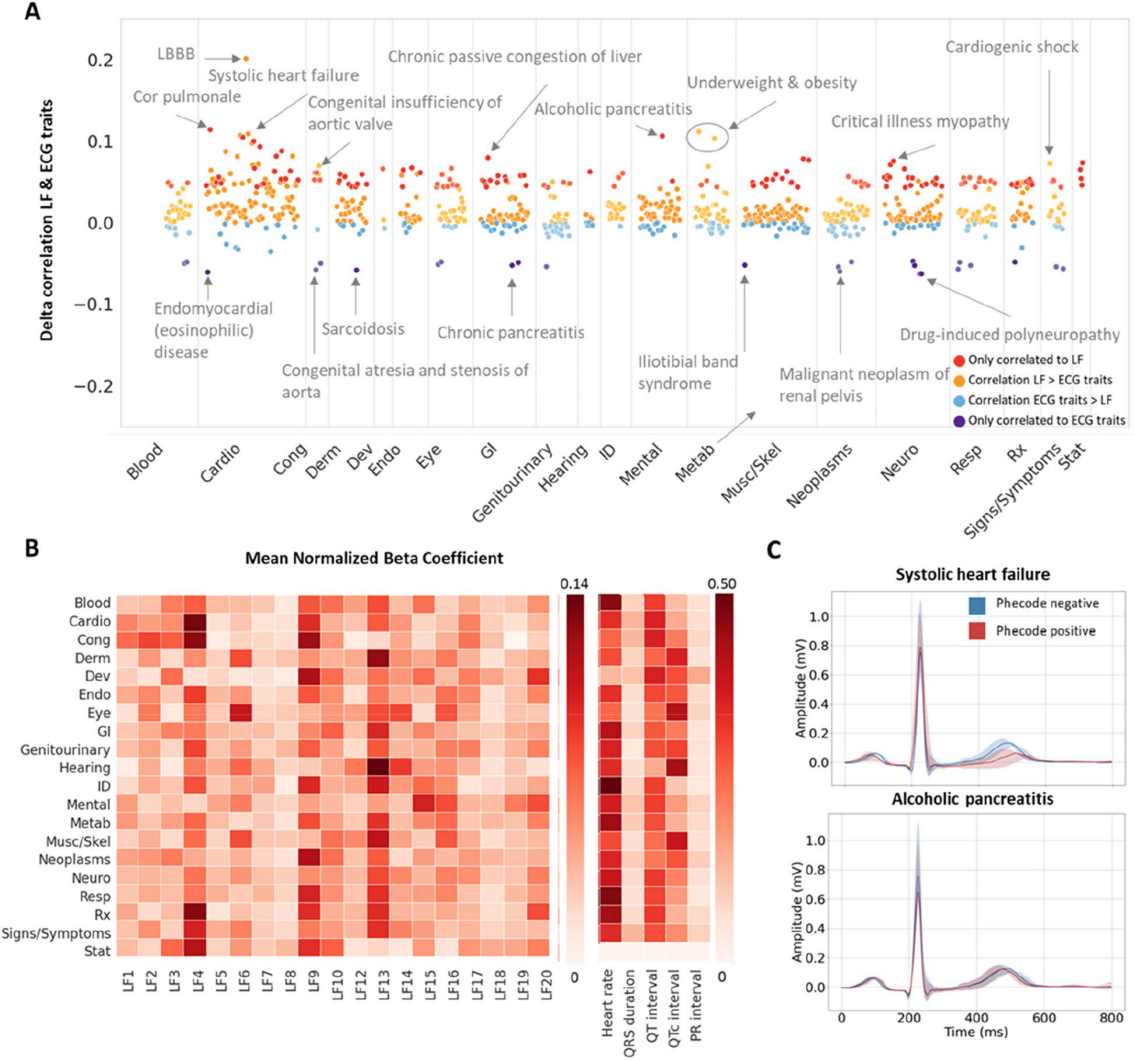
Latent factor and ECG trait associations with categorical disease labels (phecodes) in the BIDMC dataset. **A** Difference between the point biserial correlation coefficients for the actual and predicted phecode status with the model trained on latent factors (LFs) or ECG traits. Positive deflections: higher correlations to the LF predictions, negative deflections: higher correlations to the ECG traits predictions. Colour code: red, phecodes only correlated to the LF predictions; orange, higher correlations with the LF model; blue, higher correlations with the ECG traits model; purple, phecodes only correlated to the ECG trait predictions. **B** Heatmap of the category mean of the absolute beta parameter from the multivariate regression model, normalised to the sum of all beta coefficients for the category. **C** The lead I median ECG reconstructions stratified by phecode status for systolic heart failure and alcoholic pancreatits. Lines: reconstructions at the population mean, shaded area: ± 0.5 standard deviations. Category label abbreviations: Blood, blood/immune disorders; Cardio, cardiovascular disorders; Cong, congenital disorders; Derm, dermatological disorders, Neoplasms; Neuro, neurological disorders; Resp, respiratory disorders; ID, infectious diseases; Rx, disorders due to external agents (e.g. medications); Musc/Skel, musculoskeletal disorders; Mental, mental health disorders; GI, gastrointestinal disorders; Metab, metabolic disorders; Derm, dermatological disorders; Endo, endocrine disorders; Dev, developmental disorders; Cong, congenital disorders; Stat, status/other

Overall, 798 disease phecodes were significantly correlated to at least one of the model predictions. The LFs identified 147 phenotypic correlations which were not significant for the ECG traits at the Bonferroni selection threshold (Fig. 6A), whereas 25 of the correlated traits identified by the ECG parameters were missed by the LFs. For phecodes correlated to both LF and ECG traits predictions, 76% (473/626) had a higher correlation coefficient with the LF. Although this trend was observed for all phecode categories, it was most pronounced for cardiac diseases (Fig. 6A). The ECG traits models were mainly driven by the ventricular rate (Fig. 6B), while the contributions of the LFs appeared more varied and category dependent. We also provide lead I median ECG reconstructions stratified by phecode status for two associated phenotypes (systolic heart failure and alcoholic pancreatitis) as a demonstration of how the LFs can be used to enhance interpretability by visual inspection (Fig. 6C).

**Fig. 7:**
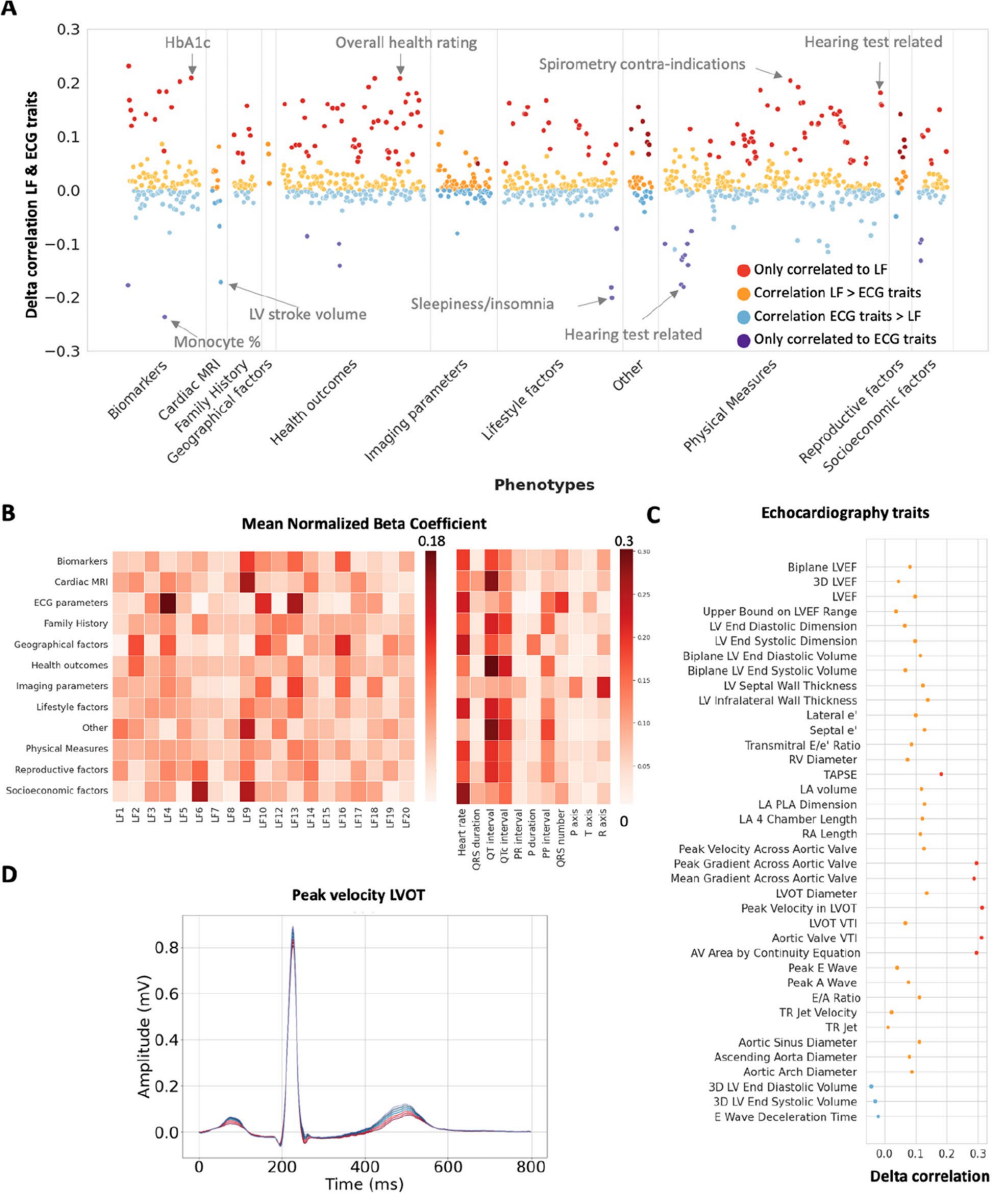
Latent factor and ECG trait associations with UK Biobank quantitative phenotypes and echocardiographic traits in the BIDMC. **A** Difference between the Pearson correlation coefficients for the actual and predicted continuous phenotypes in the UKB with the model trained on latent factors (LFs) or ECG traits. Positive deflections: higher correlations to the LF predictions, negative deflections: higher correlations to the ECG traits predictions. **B** Heatmap of the category mean of the absolute beta parameter from the multivariate regression model, normalised to the sum of all beta coefficients for the category. **C** Difference between the Pearson correlation coefficients for the actual and predicted echocardiographic traits in the BIDMC with the model trained on latent factors (LFs) or ECG traits. Colour code (**A** and **C**): red, phecodes only correlated to the LF predictions; orange, higher correlations with the LF model; blue, higher correlations with the ECG traits model; purple, phecodes only correlated to the ECG trait predictions. **D** The lead I median ECG reconstructions based on the mean ± 1–3 standard deviations values of left ventricle outflow tract (LVOT) peak velocity. Red lines indicate negative deviations, while blue lines indicate positive deviations

Similar findings were observed for the continuous phenotypes in the UKB (Fig. 7A). Out of 1083 phenotypes which were significantly correlated to at least one of the model predictions, 158 were only identified with the LFs, whereas 20 were only correlated to the ECG traits predictions. Out of the remaining phenotypes, 58% (522/905) were more highly correlated with the LF predictions. Both heart rate and the QT(c) interval proved to be the main contributors to the ECG trait models across most categories (Fig. 7B).

The difference between the LF and ECG traits associations was most pronounced for the echocardiographic traits (Fig. 7C). We found that all 35 out of the 38 traits were more highly correlated to the LF predictions, with six traits only identified by the LF model and none only by the ECG traits. The advantage of the LF model was most pronounced for traits associated with the aortic valve and the left ventricular outflow tract (Fig. 7C, D).

## Discussion

Traditionally, the interpretation of the ECG relies on the identification and measurement of human-defined morphologies and intervals. While the study of these conventional ECG parameters has yielded much insight into cardiac physiology and pathophysiology, these methods fail to fully capture the complexity of the ECG. The VAE model offers a novel approach which extracts features that more fully characterise the ECG, without anchoring on previous knowledge and concepts, and preserves high interpretability through direct signal visualisation.

While the architecture of our VAE model was overall similar to previous approaches using a convolutional network, the strength of our approach lies in the diversity of the dataset used for training our model. Our VAE model was trained on a large (> 1 million ECGs) secondary care dataset, derived from both in- and outpatient settings, to capture a broad range of pathological and normal ECGs. By use of the open-source BRAVEHEART software, we provide a median beat extraction method which is easily transferable, as it does not rely on proprietary algorithms and can be applied to different types of digital ECG file formats. Additionally, our model was able to capture a high degree of electrophysiological variability while preserving feature disentanglement. This was demonstrated in external validation in the UKB, where our model achieved a Pearson correlation coefficient of 0.95, surpassing the 0.88 obtained by previous models on the same dataset [6].

While some latent factors correspond closely to conventional ECG features (e.g. LF1 with the PR interval), others appear to capture more complex or subtle aspects of ECG morphology that may not be appreciated by visual inspection, or are not readily quantifiable using standard measurements. These may include interactions between features across different leads, dynamic waveform shapes, or features that are visually apparent but lack established definitions, such as slurring, notching, or subtle repolarisation changes.

The main novelty of our study lies in the systematic comparison between VAE-derived ECG features and conventional ECG parameters, demonstrating that the VAE approach provides a more powerful and automated representation for genetic discovery, while maintaining high interpretability. By examining the correlations and genetic associations of the 20 VAE-derived LFs and the 11 conventional ECG parameters, we were able to show that the LFs capture the majority of the phenotypical and genetic aspects of the conventional metrics. Additionally, the LFs were able to identify additional ECG characteristics and genetic associations. In a head-to-head comparison on a single dataset, while most genetic associations with the conventional ECG parameters were captured by the LF, 42% of the LF hits eluded detection by the ECG parameters.

Overall, we observed lower correlation coefficients between the LF themselves and the LFs and conventional ECG features, compared to their genetic correlation. Many genetic loci were found to associate with multiple phenotypically uncorrelated ECG features. These findings are indicative of the presence of pleiotropic genetic factors which act on multiple apparently unrelated ECG features.

The LF were able to capture genetic associations with many well-known modifiers of cardiac electrophysiology (e.g. *SCN5A*, *KCNQ1*, *NOS1AP*, *KCNH2*) as well as myocardial structure and contractile function (e.g. *TTN*, *MYH6*). Moreover, our study expanded the genetic landscape of ECG associations by identifying and validating six GWAS loci not previously linked to the ECG, despite utilising a more moderate sample size compared to many prior GWAS of ECG parameters. Two of the six prioritised genes in these loci (*NRP1* and *TRIOBP*) are associated with the same cellular component ontology terms as the previously validated GWAS genes from our study (e.g. *KCNQ1, SCN5A,* and *MYH6-7*). Five of the six novel genes have previously been implicated in diverse cardiovascular processes. These novel findings may guide further mechanistic and therapeutic exploration into these genes. The *NRP1* gene (Neuropilin-1) has been linked to cardiac regeneration in zebrafish [26]. The *TRIOBP* (TRIO and F-actin Binding Protein) gene is involved in the modulation of the assembly of the actin cytoskeleton [27]. *TRIOBP*-*1*, a splicing isoform of *TRIOBP*, has been previously shown to interact with the cardiac potassium channel *KCNH2* [28], with a potential effect on cardiac repolarisation. In our study, *TRIOBP* associated with LF1, which is closely associated to the PQ-interval, potentially identifying a novel role in cardiac electrophysiology for this gene.

The *EFEMP1* gene encodes fibulin-3, an extracellular membrane glycoprotein, expressed in many human tissues including the heart [29]. This gene is upregulated in heart failure, and recent studies in murine models have demonstrated a role in cardiac remodelling following ischaemic injury [30]. Neural precursor cell expressed developmentally down-regulated 9 (*NEDD9*) has been previously linked to endothelial fibrosis and pulmonary arterial hypertension [31]. *GPC6*, a member of the glypicans family of evolutionary conserved heparan sulphate proteoglycans anchored to the extracellular leaflet of the cell membrane, has been associated with heart failure in clinical and murine studies [32].

Apart from the common variant associations, we were also able to identify seven genes with rare variant-based associations with the LFs. Two of these (*MYBPC3* and *SCN5A*) are well-established contributors to cardiomyopathy [33] and cardiac electrophysiology [34]. The “never in mitosis A” related kinase 6 (*NEK6*) gene has been previously linked to hypertrophic cardiomyopathy (HCM) [35] and cardiac fibrosis [36] in mouse models, but had not previously been connected to human disease or the ECG. The interleukin 17 receptor A gene (*IL17RA*) encodes a membrane glycoprotein that binds proinflammatory cytokines. *IL17RA* has also been associated with inflammatory dilated cardiomyopathy [37] and heart failure [38].

The *NME7* gene belongs to a family of nucleoside diphosphate kinases with a role in ciliary transport. Common variants in the *NME7* locus have been previously linked to the QT interval [39], although the causal gene had not yet been identified. The *CCT8* gene encodes a subunit of the chaperone complex involved in the folding of actin and tubulin [40]. Common variants in the *CCT8* locus have recently been linked to HCM [41]. The *ADAMTS6* gene encodes a protease involved in the regulation of extracellular matrix composition [42]. Coding variants in the *ADAMTS6* gene have been linked to the QRS interval [43].

Further validation of these novel findings through functional studies could expand our understanding of their impact on cardiac electrophysiology. Ideally, such functional studies would involve both a cellular model, such as induced pluripotent stem cell-derived cardiomyocytes (iPSC-CMs), which closely reflects human genetic architecture [44], and an animal model, such as zebrafish [45], to capture organ- and organism-level modifier effects. Although functional studies have historically been constrained by high costs and labour-intensive processes, recent advancements in high-throughput validation platforms [46] offer promising new avenues for streamlined assessment.

Apart from genomic associations, the VAE-derived features also proved a more powerful tool for the identification of phenotypic associations, especially for cardiac disease phecodes and echocardiographic traits. For these analyses, the LF both enabled the discovery of novel associations, otherwise overlooked by conventional ECG features, and boosted the power of known association testing. Latent factors have previously demonstrated their utility as predictive parameters in diagnostic and predictive models for cardiac disorders. By uncovering novel associations with unexpected phenotypes, we demonstrate that ECG data can extend beyond cardiac diagnoses, serving as a valuable tool for developing diagnostic and predictive models for a wide range of conditions, including non-cardiac disorders. Additionally, we demonstrated that LF visualisations can provide a clear indication of the ECG changes associated with the relevant genotypes and phenotypes. The ability to easily visualise the genetic and phenotypic associations (also made available interactively at https://www.cardiodb.org/decg_explorer/) highlights the potential of the VAE approach for the generation of new hypotheses. This approach can facilitate the discovery of novel phenotypes and guide further genetic and mechanistic explorations.

In conclusion, our study addresses limitations of traditional ECG interpretation by introducing a novel approach using the VAE. Our model, trained on a large, diverse dataset, enhances interpretability, but also surpasses previous methods in capturing variability and identifying associations for common and rare-variant genetic variants and phenotypical traits. These findings contribute valuable insights into cardiac electrophysiology, emphasising the potential of advanced analytical methods like the VAE for unravelling the complexity of the ECG.

## Conclusions

This study demonstrates the power of a novel variational autoencoder (VAE) model in uncovering new insights into cardiac electrophysiology and its genetic underpinnings. By analysing a large and diverse dataset of ECGs, we extracted interpretable latent factors that not only capture complex ECG morphology with high accuracy but also outperform traditional features in identifying genetic and phenotypic associations. These latent factors revealed novel genomic loci and rare-variant associations linked to cardiac function and structural traits, underscoring the potential of this method to advance precision medicine in cardiovascular research.

The importance of this study lies in its ability to bridge the gap between advanced machine learning approaches and interpretability, offering a practical and transparent framework for clinical and research applications. By identifying novel genetic associations and providing visualisations of their impact on ECG morphology, this approach paves the way for deeper biological insights, improved diagnostic tools, and personalised therapeutic strategies. Future work could expand upon these findings by applying this model to other populations and exploring its utility in predicting a wider range of clinical outcomes.

## Data Availability

The summary statistics supporting the GWAS findings will be made publicly available through the GWAS Catalog upon publication (GCST90624600-GCST90624630). The code used to perform the analyses and generate the plots for this is accessible in the supplement. All UKB data used in this study is publicly available to registered researchers (https://www.ukbiobank.ac.uk/). The LF generated from the UKB ECGs will be made available as a Returned Dataset in the UKB. The BIDMC dataset is restricted due to ethical limitations. Researchers affiliated to educational, or research institutions may make requests to access the datasets. Requests should be made to the corresponding author of this paper. They will be forwarded to the relevant steering committee.

## Appendix

Additional file 1: This file contains the supplementary methods, figures, and tables. Additional file 2: GWAS regions. This file contains the significant regions from the LF GWAS analysis, including the prioritised genes based on the different selection methods and the top SNP for each region. Additional file 3: Conditional SNPs. This file contains the conditionally independent genetic variants associated to the LFs; these were identified using a chromosome-wide stepwise conditional-joint analysis implemented in the GCTA software (as described in the manuscript). Additional file 4: GWAS regions tECG missed by LF. This file contains the significant regions from the traditional ECG parameter GWAS which were not identified by the LFs, including the. Additional file 5: GWAS catalog. This file describes the groupings of the GWAS catalog traits and diseases into categories and groups, which were used to generate Fig. 3 in the Manuscript. Additional file 6: Lead SNPs. This file contains the lead SNPs for each of the LF GWAS. Additional file 7: Polyfun likely causal variants: this file contains the likely causal variants for each genomic region associated with the LFs in the GWAS analysis. These variants were used for downstream annotation with the nearest protein-coding gene and gene prioritisation. Additional file 8: Polyfun likely causal variants FDR. This file contains the additional likely causal variants for the genomic regions selected at the relaxed significance threshold of 5% FDR. Additional file 9: GWAS regions FDR. This file contains the significant regions from the LF GWAS analysis at the relaxed significance threshold of % FDR, including the prioritised genes based on the different selection methods and the top SNP for each region. Additional file 10: This file contains the code used to generate this paper.

## Abbreviations

BIDMC: Beth Israel Deaconess Medical Center
BMI: Body mass index
BRAVEHEART: Beth Israel Analysis of Vectors of the Heart
ECG: Electrocardiogram
FDR: False discovery rate
FUMA: Functional Mapping and Annotation of Genome-Wide Association Studies
GCTA: Genome-wide complex trait analysis
GREML: Genomic-relatedness-based Restricted Maximum Likelihood
GWAS: Genome-Wide Association Study
HCM: Hypertrophic cardiomyopathy
ICD: International Classification of Diseases
iPSC-CMs: Induced pluripotent stem cell-derived cardiomyocytes
KL: Kullback-Leibler
LF: Latent factor
LV: Left ventricle
LVOT: Left ventricular outflow tract
MRI: Magnetic resonance imaging
NEDD9: Neural Precursor Cell Expressed Developmentally Down-Regulated 9
PoPS: Polygenic Priority Score
PIP: Posterior inclusion probability
QTSCD: QT Interval and Sudden Cardiac Death
REGENIE: A tool for whole-genome regression in genetic studies
SD: Standard deviation
SNP: Single nucleotide polymorphism
UKB: UK Biobank
VAE: Variational autoencoder
V2G: Variant-to-gene
WES: Whole-exome sequencing
