# Supplementary material for: Unsupervised feature extraction using deep learning empowers discovery of genetic determinants of the electrocardiogram
Source: Genome Med. 2025 Oct 9;17:118. doi: 10.1186/s13073-025-01510-z (PMC12512816; doi:10.1186/s13073-025-01510-z)
Supplement: Supplementary file 1 — Additional file 1: This file contains the supplementary methods, figures, and tables. [file 13073_2025_1510_MOESM1_ESM.docx]

**Supplementary methods**

**Ethical approvals**

For the Beth Israel Deaconess Medical Center (BIDMC) cohort ethics review and approval was provided by the Beth Israel Deaconess Medical Center Committee on Clinical Investigations, IRB protocol # 2023P000042.

The UK Biobank has approval from the North West Multi-Centre Research Ethics Committee as a Research Tissue Bank (application IDs 48666, 47602).

**ECG datasets**

*The BIDMC cohort*

The BIDMC cohort is a dataset comprised of routinely collected data from Beth Israel Deaconess Medical Center, Boston, USA. Subject over 16 years old with a valid ECG performed from 2014 to 2023 were included. Prior ECGs back to 2000 were included for these subjects. Diagnostic International Classification of Diseases (ICD) codes were used to determine disease status. Subjects were censored at time of outcome or last in-person hospital contact.

*The UK Biobank Cohort*

The UK Biobank is longitudinal study of over 500,000 volunteers aged 40-69 at the time of enrolment in 2006-2010. At baseline assessment participants provided information on health and lifestyle via questionnaire, had physical measures taken (including height, weight, and blood pressure) and donated samples of blood urine and saliva. A subgroup of participants were invited back for subsequent visits for additional investigations, including for detailed studies including cardiac magnetic resonance imaging (MRI), brain MRI and digital ECGs. 42,386 subjects with digital ECGs taken at the instance 2 visit were available for the initial analysis, an additional 24,355 individual were included in the validation tranche. Outcomes were linked to cancer and death registry data, hospital admissions and primary care records.

The subsets of the BIDMC and the UKB datasets used for the different tasks described in the manuscript are summarised in the flow chart below.


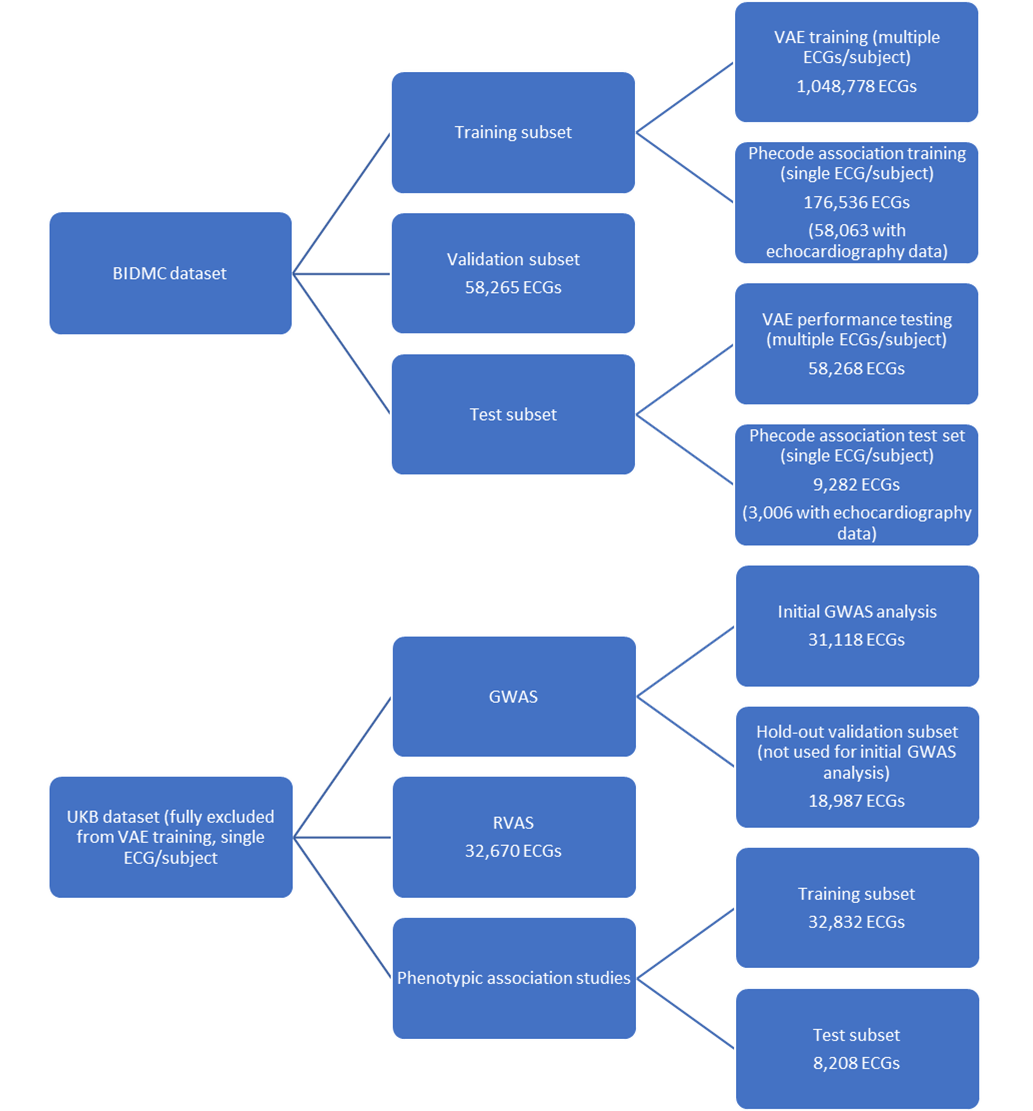


**Variational Autoencoder (VAE) architecture and training**

*
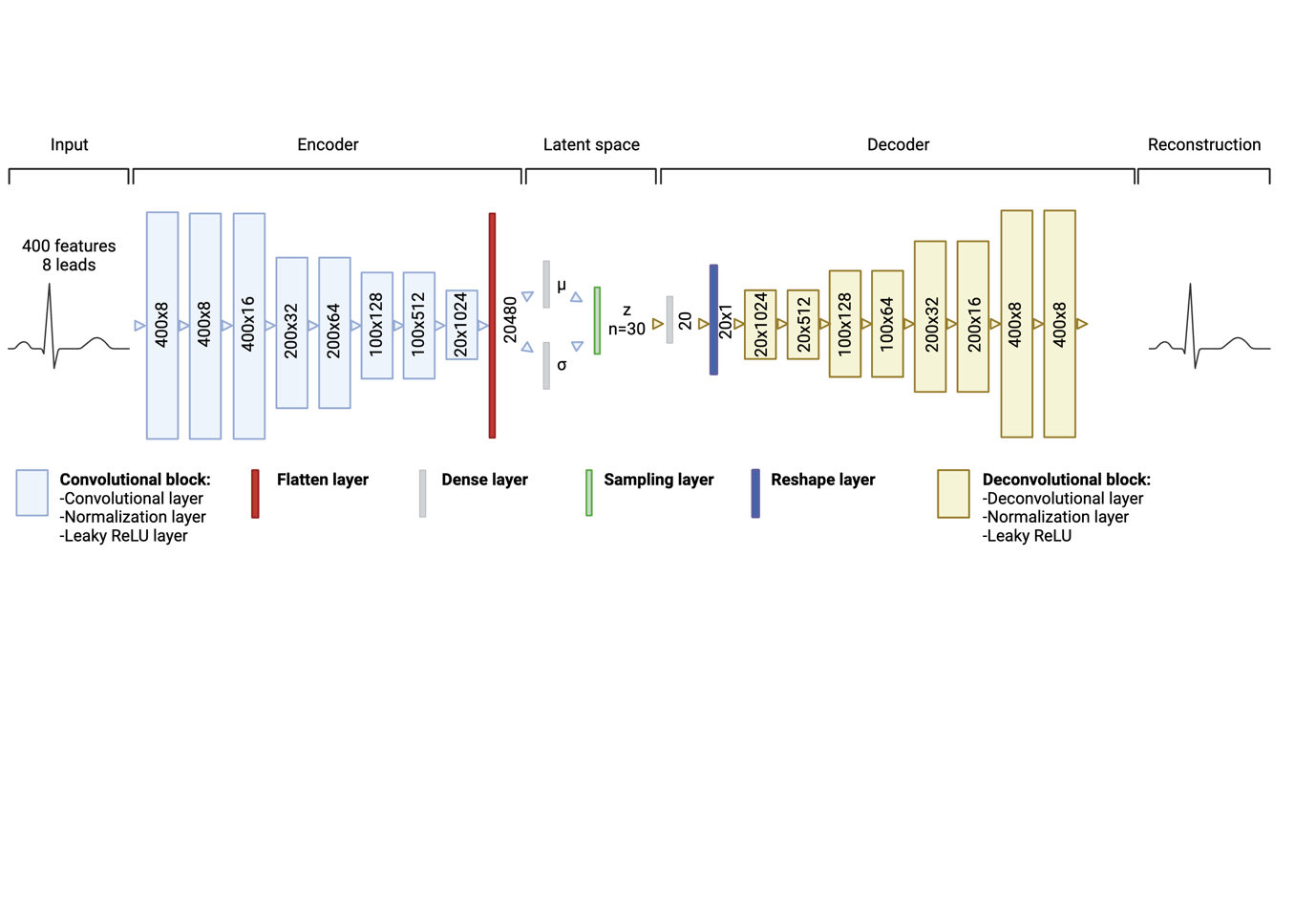
*

*Fig. S1: Variational Autoencoder (VAE) architecture*

The model was trained at a learning rate of 0.001 for 100 epochs. The optimal model was selected at the epoch with the lowest reconstruction loss (epoch 61 for the final model).

**Gene prioritisation algorithm**


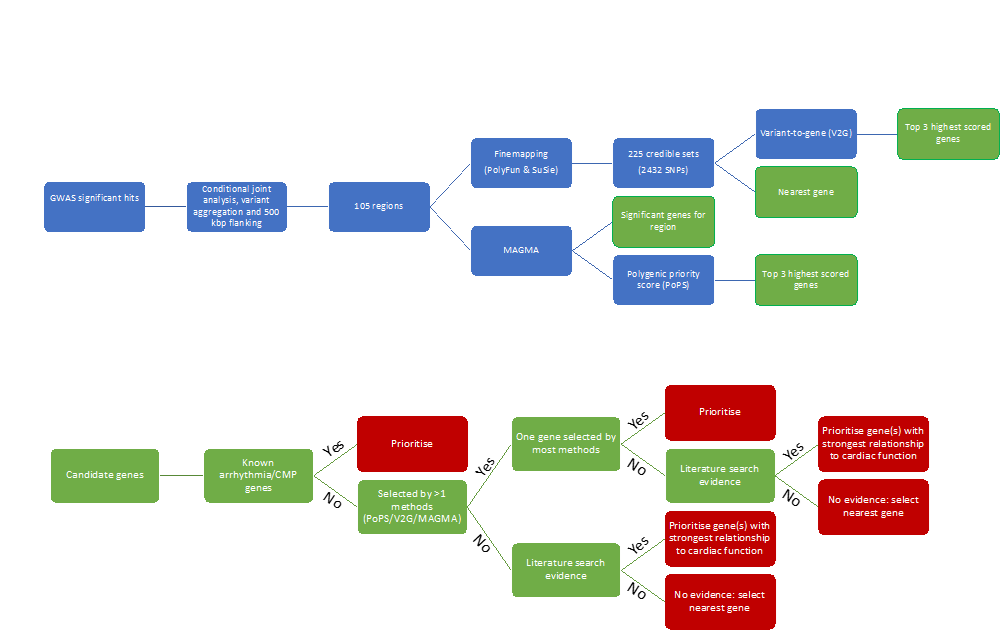


*Fig. S2: Gene prioritisation algorithm for GWAS loci.* Blue: from GWAS significant hits to candidate genes, green: from candidate genes to prioritised genes, red: prioritised genes

**Individual latent factor data**

Full 8-lead latent factor traversal plots, individual latent factor GWAS Manhattan plots and heritability calculation results are available through an interactive web service <https://www.cardiodb.org/decg_explorer/>. These plots were made based on individual LF GWAS associations and mark all associations at the conventional genome wide significance threshold of P < 5 x 10^-8^ with the mapped gene. A reverse approach mapping the associations of the identified gene to the ECG is available the same site as well as an interactive 8-lead median beat reconstruction plot.

**Genetic correlation with age**

| LF | rg age |
| --- | --- |
| 1 | -0.13 |
| 2 | 0.09 |
| 3 | -0.03 |
| 4 | 0.12 |
| 5 | -0.15 |
| 6 | -0.01 |
| 7 | 0.10 |
| 8 | -0.12 |
| 9 | -0.13 |
| 10 | 0.20 |
| 11 | -0.26 |
| 12 | -0.37 |
| 13 | -0.01 |
| 14 | -0.14 |
| 15 | -0.04 |
| 16 | -0.04 |
| 17 | 0.15 |
| 18 | -0.13 |
| 19 | -0.31 |
| 20 | 0.15 |

*Table S1: Genetic correlation with age. To assess the specificity of the LF correlations and provide a negative control, we also evaluated genetic correlations between the 20 LFs and age (a non-ECG trait). This table depicts the genetic correlations between latent factors and age in the UKB (calculated as described for the other traits in the Methods section).*

**Rare variant association study (gene burden testing) result**

| Latent Factor | Chromosome | Gene | Allele freq | N | Mask | Beta | SE | ChiSQ | log 10 p-value |
| --- | --- | --- | --- | --- | --- | --- | --- | --- | --- |
| 19 | 9 | *NEK6* | 0.0002 | 32670 | Singleton | 1.46 | 0.31 | 22.67 | 5.72 |
| 5 | 22 | *IL17RA* | 0.0002 | 32670 | Singleton | -1.28 | 0.26 | 24.43 | 6.11 |
| 7 | 1 | *NME7* | 0.0003 | 32670 | Singleton | -0.96 | 0.22 | 18.46 | 4.76 |
| 10 | 11 | *MYBPC3* | 0.0002 | 32670 | <0.001 | -1.21 | 0.29 | 17.17 | 4.47 |
| 11 | 21 | *CCT8* | 0.0005 | 32670 | <0.001 | 0.65 | 0.17 | 14.29 | 3.81 |
| 3 | 5 | *ADAMTS6* | 0.0065 | 32670 | <0.01 | 0.18 | 0.04 | 15.72 | 4.13 |
| 1 | 3 | *SCN5A* | 0.0061 | 32670 | <0.01 | -0.18 | 0.05 | 14.01 | 3.74 |

*Table S2: Genes significantly associated with the latent factors at the FDR <5% significance threshold*

|  | **Minor allele frequency** | | | | |
| --- | --- | --- | --- | --- | --- |
| **Beta** | **0,1** | **0,2** | **0,3** | **0,4** | **0,5** |
| **0,05** | 0,01 | 0,17 | 0,40 | 0,56 | 0,61 |
| **0,0875** | 0,72 | 1 | 1 | 1 | 1 |
| **0,125** | 1 | 1 | 1 | 1 | 1 |
| **0,1625** | 1 | 1 | 1 | 1 | 1 |
| **0,2** | 1 | 1 | 1 | 1 | 1 |

*Table S3: GWAS power calculation at p-value of 5 × 10−8/20 in a sample set of 31,118 individuals (calculated with method described in Visscher PM, Wray NR, Zhang Q, et al. 10 Years of GWAS Discovery: Biology, Function, and Translation. Am J Hum Genet 2017;101(1):5-22. doi: 10.1016/j.ajhg.2017.06.005)*
